# Concurrent Intraductal Papilloma in a Patient with Nipple Adenoma

**DOI:** 10.51894/001c.165611

**Published:** 2026-07-31

**Authors:** Sara Glendinning, Angelo Federico, Helen Mabry

**Affiliations:** 1 Corewell Dearborn/Trenton

## 85

### Introduction

Nipple adenoma is a rare benign proliferative lesion that makes up less than 1% of diagnosed benign breast lesions. The incidence is highest in women in the 4th-5th decade of life. The diagnosis of an intraductal papilloma is more common and represents an estimated 2-3% of all benign breast tumors. Intraductal papilloma has a highest incidence in women in the 3rd-5th decade of life. The concomitant diagnoses of these pathologies is extremely rare and the available literature regarding a simultaneous diagnosis is very limited.

### Case Description

We report the case of a 34-year-old female that presented with a left-sided nipple lesion discovered on self-exam. She noticed the lesion two years prior and endorsed a steady increase in size over time. On examination, the 1.1cm lesion was flesh-colored and located in the center of the nipple. No lymphadenopathy or skin dimpling was appreciated. She subsequently underwent a diagnostic mammogram which revealed a fibroglandular density and asymmetric prominence of the left nipple with a BIRADS 4A classification. Ultrasound demonstrated a 9mm complex fluid collection within the nipple. Given the BIRADS classification and cosmetic concerns, the patient opted for surgical excision. Resultant pathology revealed both nipple duct adenoma and intraductal papilloma with florid ductal hyperplasia and focal atypia.

### Discussion

Appropriate workup of nipple lesions is imperative to ensure the correct diagnosis is made and allow for the appropriate treatment. It also ensures concomitant pathologies are not missed. Workup includes a thorough history and physical exam, imaging, and ultimately surgical excision. In our case, the patient underwent both mammogram and ultrasound; neither of these modalities demonstrated radiographic evidence of an intraductal papilloma. In addition, she didn’t have pathologic symptomatology, such as bloody nipple discharge. This reinforces the need for surgical excision to confirm the suspected diagnosis, as well as rule out possible underlying pathologies. If patients choose less invasive diagnostic approaches, the possibility of underlying pathology must be discussed. Our case above highlights the rare occurrence of concomitant nipple adenoma and intraductal papilloma and brings attention to the need for a complete workup even when benign breast pathology is suspected.

